# Prevalence of Periodontitis in Patients with Established Rheumatoid Arthritis: A Swedish Population Based Case-Control Study

**DOI:** 10.1371/journal.pone.0155956

**Published:** 2016-05-20

**Authors:** Kaja Eriksson, Lena Nise, Anna Kats, Elin Luttropp, Anca Irinel Catrina, Johan Askling, Leif Jansson, Lars Alfredsson, Lars Klareskog, Karin Lundberg, Tülay Yucel-Lindberg

**Affiliations:** 1 Department of Dental Medicine, Division of Periodontology, Karolinska Institutet, Huddinge, Sweden; 2 Institute of Environmental Medicine, Karolinska Institutet, Solna, Sweden; 3 Department of Medicine, Rheumatology Unit, Karolinska University Hospital, Solna, Stockholm, Sweden; 4 Department of Periodontology at Eastmaninstitutet, Stockholm County Council, Stockholm, Sweden; 5 Centre of Environmental and Occupational Medicine, Stockholm County Council, Stockholm, Sweden; Nippon Medical School Graduate School of Medicine, JAPAN

## Abstract

**Introduction:**

The possible hypothesis of a link between periodontitis and rheumatoid arthritis (RA), specifically anti-citrullinated protein antibody (ACPA) positive RA, prompted us to investigate the prevalence of periodontitis in the Swedish Epidemiological Investigation of RA (EIRA), a well-characterised population-based RA case-control cohort.

**Methods:**

Periodontal status of 2,740 RA cases and 3,942 matched controls was retrieved through linking EIRA with the National Dental Health Registry (DHR), where dental diagnostic- and treatment codes on the adult Swedish population have been registered. Dental records from 100 cases and controls were reviewed to validate the periodontal diagnostic codes in DHR.

**Results:**

The reviewed dental records confirmed 90% of the periodontitis diagnoses in DHR among RA cases, and 88% among controls. We found the positive predictive value of periodontitis diagnoses in the DHR to be 89% (95% CI 78 to 95%) with a sensitivity of 77% (95% CI: 65 to 86%). In total, 86% of EIRA participants were identified in DHR. The risk for periodontitis increased by age and current smoking status in both cases as well as controls. No significant differences in prevalence of periodontal disease in terms of gingivitis, periodontitis, peri-implantitis or increased risk for periodontitis or peri-implantitis were observed between RA cases and controls. In addition, there was no difference on the basis of seropositivity, ACPA or rheumatoid factor (RF), among patients with RA.

**Conclusions:**

Our data verify that smoking and ageing are risk factors for periodontitis, both in RA and controls. We found no evidence of an increased prevalence of periodontitis in patients with established RA compared to healthy controls, and no differences based on ACPA or RF status among RA subjects.

## Introduction

Rheumatoid arthritis (RA) and periodontitis are multifactorial complex diseases characterized by common pathogenetic mechanisms of chronic inflammation and bone destruction [1–4]. In addition, these two widespread diseases share a number of risk factors, most notably smoking [1–3, 5–8]. The systemic disease RA, an autoimmune disorder with unclear etiology, is characterized by synovial joint inflammation and pannus formation leading to irreversible destruction of cartilage and underlying bone [1, 9]. Periodontitis, on the other hand, is an immunoinflammatory disease initiated by oral pathogens and characterized by a continuous inflammatory reaction leading to destruction of the supporting structures around the teeth [10, 11]. Previous observational studies based on clinical cohorts have suggested that the prevalence of RA is higher in patients with periodontitis than in patients without periodontitis and vice versa, i.e. indicating that patients with RA may have an increased frequency of periodontitis/severe periodontitis compared to controls [12–19]. Several other reports have, however, failed to replicate these findings, reporting no association between periodontitis and RA or even less severe periodontal tissue destruction in subjects with RA [20–26].

The results from population-based studies on the potential connection between periodontitis and RA are inconsistent [21, 27–29]. In the largest prospective study so far (81 132 participants including 292 incident RA cases), conducted by Arkema et al, no association was found between severe periodontitis, estimated by history of periodontal surgery and/or tooth loss, and risk of RA [21]. Also, Demmer et al [29], in a study of 9702 participants with 138 prevalent and 433 incident RA cases, reported non-significant higher odds of prevalent/incident RA in patients with periodontitis compared to controls. In contrast, de Pablo et al reported that patients with RA were more likely to be totally edentulous and have periodontitis compared to non-RA subjects [28]. Likewise, the largest study to date, a nationwide study in Taiwan (13 779 RA cases and 137 790 controls) described a weak association between periodontitis and incident newly diagnosed patients with RA [27]. However, the studies mentioned above, both clinical and large register-based, were limited due to either lack of individual smoking status, small numbers of subjects with RA and/or the lack of uniformity in the definition of periodontitis. In addition, none of the population-based studies have included information on anti-citrullinated protein antibody (ACPA) status, which has been associated with bone loss [30].

Presence of ACPA indicates a more severe and destructive disease phenotype [1, 31] and a strong gene-environment interaction between smoking and *HLA-DRB1* shared epitope (SE) alleles in the development of RA has been shown only for ACPA-positive disease [32, 33]. Interestingly, ACPA have been detected in sera and in gingival crevicular fluid in non-RA subjects with periodontal disease, although at very low levels [34–36]. During the last decade, a hypothesis potentially explaining an association between RA and periodontitis, involving the periodontal pathogen *Porphyromonas gingivalis* (*P*.*gingivalis*) has emerged [6]. This periodontal pathogen is the only reported bacteria able to express the enzyme peptidylarginine deiminase (PAD) with the capacity to generate citrullinated proteins and peptides, which may trigger autoimmune response in RA [37]. Therefore, when investigating the potential relationship between periodontitis and RA, ACPA status could be an important factor to be considered.

In summary, although previous studies indicate a potential association between RA and periodontitis, the strength and temporality of this association is still unclear due to the lack of unbiased well-defined population based epidemiological studies. Thus, further research is warranted to clarify the relationship between RA and periodontitis [8, 38–40]. Our aim was therefore to investigate the prevalence of periodontitis amongst patients with RA, with special focus on seropositivity, both ACPA and rheumatoid factor (RF), in the Swedish population-based Epidemiological Investigation of Rheumatoid Arthritis (EIRA) case-control study, by linking EIRA with the National Dental Health Registry (DHR).

## Methods

### Study population

**Fig 1:**
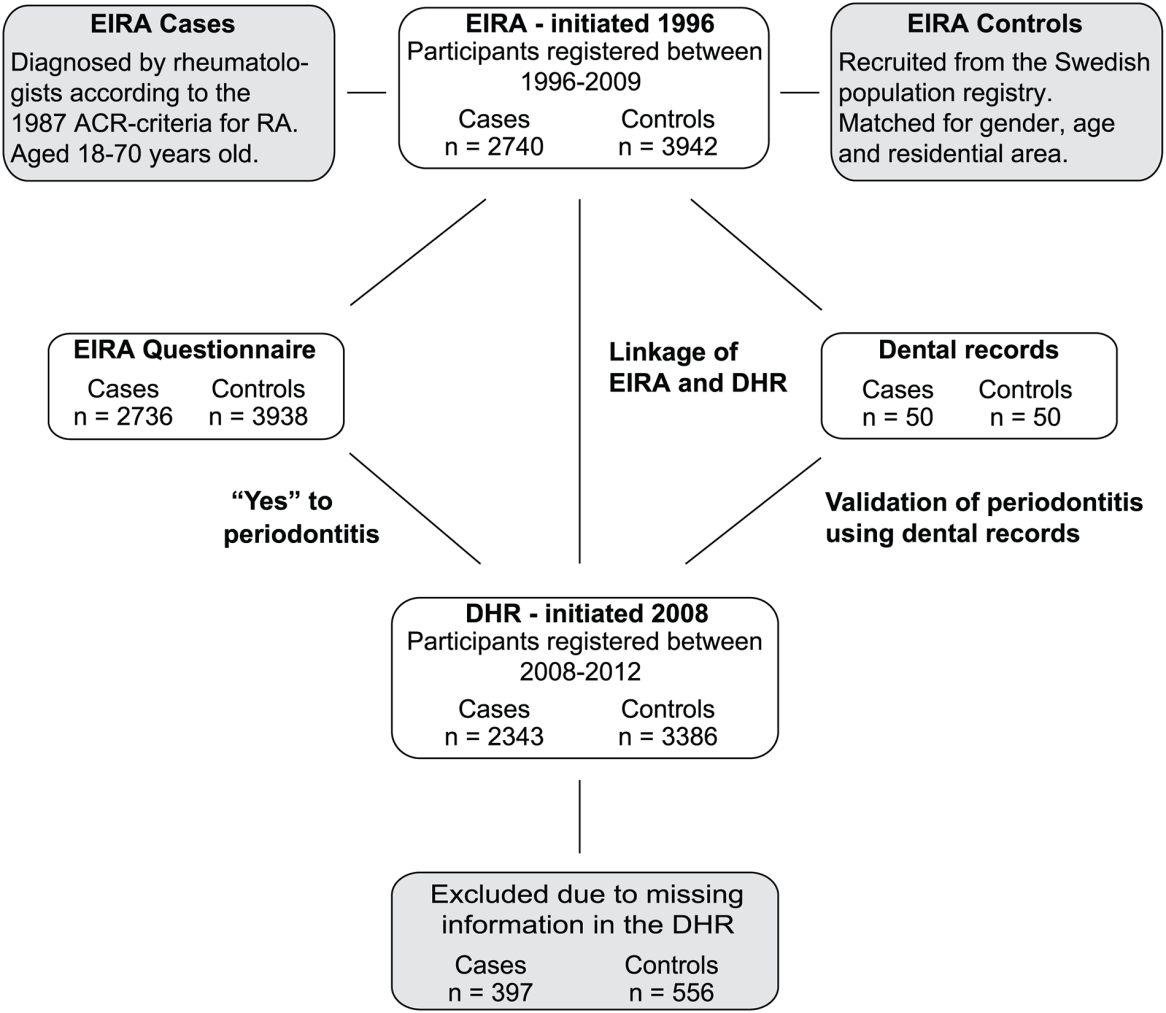
Study design. Schematic overview of the study, including linkage of EIRA (Epidemiological Investigation of Rheumatoid Arthritis) with DHR (Dental Health Registry) and the validation of the diagnostic codes from DHR using dental records.

The study included 6682 participants; 2740 subjects diagnosed with RA and 3942 matched controls enrolled from the Swedish population-based case-control study EIRA. Prevalence of periodontitis amongst participants was investigated using a self-administered questionnaire, given to patients with RA shortly after diagnosis at time of recruitment into EIRA, linkage analysis between the EIRA registry and DHR as well as patients dental records (Fig 1).

### EIRA case-control study

EIRA consists of incident cases of RA and matched controls aged 18–70. The study was initiated in May 1996, and the present investigation includes participants until October 2009 [33, 41, 42]. All cases were newly diagnosed RA patients and diagnosis was confirmed by rheumatologists according to the 1987 American College of Rheumatology Criteria for RA [43] at the time of their enrolment. Controls were randomly obtained from the Swedish national population registry and matched to cases on age at diagnosis (±2.5 years), gender and residential area. Blood serum samples were collected at the time of recruitment, and information about the serological markers of RA, ACPA (as determined by the CCPlus^®^ assay) and RF status determined for cases and for some controls [33]. Participants completed a questionnaire at their recruitment, at the time of their RA diagnosis, and the controls were collected over the same period as the actual cases. Questions covered a broad range of topics, including smoking habits and one question related to periodontal disease: “Have you had infection of the teeth or gums (periodontitis / root infection)?”.

### Dental Health Registry (DHR)

The Swedish National DHR, initiated in July 2008 to follow the development of dental care, consists of diagnostic and treatment codes for numerous dental and orofacial conditions in Swedish citizens over the age of 20. Like all the Swedish registries, DHR is based upon patients’ national social security numbers and information can therefore be linked to other health data registries in Sweden. Whenever a patient visits the dentist, a treatment code in combination with a diagnostic code is registered by the dentist and this code is used for reimbursement purposes. All data is reported to the National Board of Health and Welfare (Socialstyrelsen) and included in the DHR database.

### Linkage analysis of EIRA and DHR

Data from EIRA was linked with DHR using unique social security numbers. Diagnostic codes and treatments codes related to periodontal disease were requested from DHR and a dataset of anonymized participants was received. Codes not generally used by dentists and/or containing other non-periodontal treatment causes were used only when analysing “Any diagnosis” and were not included in further analysis. The investigated diagnostic codes from DHR were: increased risk for periodontitis (2041); increased risk for peri-implantitis (2051); gingivitis (3041); periodontitis (3043) and peri-implantitis (3044). Diagnostic codes excluded from further analysis due to not being generally used by dentists and/or containing other non-periodontal treatment causes were: tooth defect/loss of tooth substance when evaluating cariological, physiological or periodontal treatment needs before permanent rehabilitation (4883); partial edentulism in position 5–5 when evaluating cariological, physiological or periodontal treatment needs before permanent rehabilitation (5045); periodontally damaged dentition with prosthetic needs without previous periodontal treatment (5061) and periodontally damaged dentition with prosthetic needs with previous treatment for periodontal disease (5062). To investigate the severity of periodontitis, the diagnostic codes for periodontitis and increased risk for periodontitis were also analysed with respect to periodontal treatment. Treatment codes investigated in this study were: minor non-surgical treatment of periodontal disease (341); major (including several deep pockets and/or furcation involvement) non-surgical treatment of periodontal disease (342) and surgical treatment of teeth affected by periodontitis (441 and 442).

### Dental records

To validate periodontitis diagnosis from DHR, dental records were requested from some EIRA patients responding “Yes” to the periodontitis-related question in the EIRA questionnaire. Requests were sent to 151 subjects with RA and 155 controls, of whom 50 subjects respectively gave their written informed consent to have their dental records reviewed. Diagnosis obtained by codes in DHR was compared to diagnosis using dental radiographs and additional information in the patient dental records. All information in the dental records, including all radiographs, was examined by two independent dentists. Periodontal diagnosis was set by following criteria of the International Consensus Report on Chronic Periodontitis; where extent of chronic periodontitis was classified as localized (≤30% of the sites are affected) or generalized (>30% of the sites are affected), and severity of chronic periodontitis was classified as slight (1 to 2 mm of clinical attachment loss (CAL)), moderate (3 to 4 mm CAL) or severe (≥5 mm CAL) [44]. For the validation, all patients showing any sign of chronic periodontal disease were classified as having periodontitis, independent of the extent or severity.

### Statistical analysis

EIRA participants, controls and RA cases with diagnostic codes for periodontitis were compared to subjects without periodontitis codes. Unconditional logistic regression analysis was used to estimate Odds ratio (OR) with 95% Confidence Interval (CI) and all analyses were adjusted for the matching variables gender, age, smoking habits and residential area. The ORs were interpreted as estimates of rate ratios since the study was population-based and the matched controls selected from the same study base. SAS software, version 9.2 (SAS Institute, Cary, NC) was used for statistical analysis. The differences between the groups were analysed by chi-square test or Fisher´s exact test.

### Ethics Statement

The study was performed in accordance with the Declaration of Helsinki and in accordance with the current legislation in Sweden. The Regional Ethical Review Board in Stockholm approved the study design, the linkage of the data from the registries as well as the validation of the diagnostic codes using patient’s dental records (Dnr numbers 2009/792-31/4, 2011/123-32, 2010/388-32), and written consent was obtained from all subjects.

## Results

### Characteristics of the study population

In the present study we focused our analyses on 2,343 RA cases and 3,386 gender-, age- and residential area-matched controls. These individuals represent 86% of the total EIRA study population of 6,682 individuals, and were selected based on being identified in DHR through linking of registries (Fig 1). Importantly, demographic data did not differ significantly between EIRA subjects identified in DHR and the total EIRA study population (S1 Table). In the DHR study population, 73% were women, 63% of the RA patients were ACPA-positive and 64% RF-positive. Smoking habits were comparable for RA cases and controls concerning non-regular smokers, whereas the percentage of current smokers was significantly higher among RA cases (25%) compared to controls (18%) (p < 0.0001), as previously reported [45]. Also in line with previous reports [41], having a university degree was more common among controls (31%) than among patients with RA (24%) (p < 0.0001). We also analysed the only question in the EIRA questionnaire relating to periodontal status, and of the total EIRA population (2740 RA and 3942 matched controls), 18% of RA and 16% of controls reported that they had periodontitis (S1 Table). Of the subjects identified in the DHR, 17% of RA and 16% of controls self-reported periodontitis (Table 1).

**Table 1:** Characteristics of the total EIRA study population identified in DHR.

Characteristics	RA cases (n = 2343)	Controls (n = 3386)	p-value^†^
**Gender**			
Male	624 (27)	917 (27)	NS
Female	1719 (73)	2469 (73)	NS
**Age**			
18–29 years	168 (7)	247 (7)	NS
30–39 years	280 (12)	413 (12)	NS
40–49 years	408 (17)	597 (18)	NS
50–59 years	760 (32)	1056 (31)	NS
60–70 years	727 (31)	1072 (32)	NS
**RA duration in years (SD)**	9.6 (3.9)	N/A	
**ACPA status**			
ACPA-positive	1469 (63)	N/A	
ACPA-negative	852 (36)	N/A	
**RF status**			
RF-positive	1505 (64)	N/A	
RF-negative	822 (35)	N/A	
**Smoking habits**			
Never smokers	777 (33)	1459 (44)	<0.0001
Ex-smokers	757 (32)	965 (29)	<0.02
Current smokers	576 (25)	588 (18)	<0.0001
Non-regular smokers	226 (10)	341 (10)	NS
**Education**			
University degree	567 (24)	1066 (31)	<0.0001
No university degree	1774 (76)	2307 (68)	<0.0001
**Self-reported periodontitis**			
Yes	410 (17)	526 (16)	
No	1931 (82)	2856 (84)	<0.05

Results are presented as number (%). EIRA, Epidemiological Investigation of Rheumatoid Arthritis; DHR, Dental Health Registry; RA, rheumatoid arthritis; ACPA, anti-citrullinated protein antibody; RF, rheumatoid factor; N/A, not applicable; NS, not significant; SD, standard deviation.

^†^Statistical difference between RA cases and controls in DHR. The differences between the groups were analysed by chi-square test or Fisher´s exact test.

p-value < 0.05 was considered statistically significant.

### Validation of the periodontal diagnostic codes in DHR

Dental records from 50 EIRA RA cases and 50 EIRA controls were retrieved for validation of the periodontal diagnostic codes registered in DHR (Fig 1). These 100 subjects were initially selected based on having self-reported periodontitis according to the EIRA questionnaire. Of these subjects, 48 RA cases and 49 controls were identified in DHR, but only 29 RA cases and 34 controls had periodontitis according to the diagnostic codes in DHR. Hence, self-reported periodontitis could not be validated against DHR, which is in line with previous reports on the reliability of self-reported periodontitis [46–48]. On the contrary, comparison of the DHR diagnostic codes with the dental records confirmed approximately 90% of the periodontitis diagnoses (PPV = 89%, 95% CI: 78 to 95%). The sensitivity of the DHR was 77% (95% CI: 65 to 86%), and the specificity was 71% (95% CI: 49 to 87%), based on validation against dental records (S2 Table).

### Prevalence of periodontitis in RA cases and controls in relation to ACPA and RF status

Approximately 70% of RA cases and controls, identified in DHR, had at least one of the diagnostic codes related to periodontal disease (Table 2). The most frequent diagnostic codes observed were gingivitis (33% of RA and 35% of controls) and periodontitis (33% of RA and 32% of controls). No differences in prevalence between RA and controls were found when investigating the combined diagnostic codes related to periodontal disease (including gingivitis, periodontitis, increased risk for periodontitis, peri-implantitis and increased risk for peri-implantitis) indicated as “any diagnosis” (Table 2). Furthermore, the prevalence of the individual diagnostic codes gingivitis, periodontitis, peri-implantitis or increased risk for periodontitis/peri-implantitis did not differ between RA cases and controls. When stratifying the analyses by ACPA or RF status among RA cases, no significant differences were observed (Table 2). The distribution of “missing EIRA subjects”, i.e. those that were not identified in DHR, ranged from 15% to 16% for the different diagnostic codes with no differences observed between RA and controls (S3 Table).

**Table 2:** EIRA RA cases and controls with periodontal diagnostic codes identified in DHR, in relation to ACPA and RF status.

Diagnosis	RA cases					Controls	p-value^†^
	All	ACPA-positive	ACPA-negative	RF-positive	RF-negative		RA cases vs controls ACPA/RF-positive vs negative RA cases
Any diagnosis^††^	1629 (70)	1021 (70)	591 (69)	1053 (70)	565 (69)	2381 (70)	NS
Gingivitis	784 (33)	487 (33)	291 (34)	508 (34)	270 (33)	1193 (35)	NS
Periodontitis	762 (33)	487 (33)	268 (31)	498 (33)	259 (32)	1091 (32)	NS
Increased risk periodontitis	597 (25)	360 (25)	230 (27)	383 (25)	209 (25)	932 (28)	NS
Peri-implantitis	109 (4.7)	72 (5.0)	37 (4.3)	76 (5.0)	33 (4.0)	140 (4.1)	NS
Increased risk peri-implantitis	17 (0.7)	10 (0.7)	7 (0.8)	12 (0.8)	5 (0.6)	20 (0.6)	NS

Results are presented as number (%). EIRA, Epidemiological Investigation of Rheumatoid Arthritis; DHR, Dental Health Registry; RA, rheumatoid arthritis; ACPA, anti-citrullinated protein antibody; RF, rheumatoid factor; NS, not significant.

^††^Any diagnosis indicates all the diagnostic codes related to periodontal disease: gingivitis (3041); periodontitis (3043); increased risk for periodontitis (2041); peri-implantitis (3044); increased risk for peri-implantitis (2051); and the codes including partial edentulousness and periodontally damaged dentition as described in subjects and methods (4883, 5045, 5062 and 5061).

^†^Statistical differences in prevalence of periodontal diagnostic codes between RA cases and controls, and between ACPA-positive and ACPA-negative or RF-positive and RF-negative RA cases. The differences between the groups were analysed by chi-square test or Fisher´s exact test.

p-value < 0.05 was considered statistically significant.

To further evaluate the severity of periodontitis among RA cases and controls, we also analysed the periodontal treatment codes in relation to ACPA status. The prevalence of periodontal treatment codes did not differ between RA and controls nor between ACPA-positive and ACPA-negative RA (Table 3). The prevalence of non-surgical periodontal treatment (both minor and major) was, however, higher among subjects diagnosed with periodontitis (varying between 59–61% for minor and 58–56% for major non-surgical periodontal treatment in RA cases and controls, respectively), as compared to treatment for increased risk for periodontitis (varying between 31–32% for minor and 23–22% for major in RA cases and controls, respectively). Furthermore, 2% of both RA cases and controls with periodontitis were treated surgically (Table 3).

**Table 3:** EIRA RA cases and controls with periodontal treatment codes, in relation to ACPA status.

	RA cases			Controls	p-value^†^
	All	ACPA-positive	ACPA-negative		RA cases vs controls ACPA-positive vs negative RA cases
**Treatment for Periodontitis**					
Minor non-surgical treatment	452 (59)	291 (60)	157 (59)	668 (61)	NS
Major non-surgical treatment	440 (58)	280 (57)	154 (57)	610 (56)	NS
Surgical treatment	15 (2)	12 (2)	3 (1)	24 (2)	NS
**Treatment for Increased risk for periodontitis**					
Minor non-surgical treatment	188 (31)	116 (32)	72 (31)	301 (32)	NS
Major non-surgical treatment	139 (23)	87 (24)	51 (22)	206 (22)	NS
Surgical treatment	4 (1)	3 (1)	1 (0)	9 (1)	NS

Results are presented as number (%). EIRA, Epidemiological Investigation of Rheumatoid Arthritis; RA, rheumatoid arthritis; ACPA, anti-citrullinated protein antibody; NS, not significant.

^†^Statistical significance for differences in treatment between RA cases and controls, and between ACPA-positive and ACPA-negative RA cases. The differences between the groups were analysed by chi-square test or Fisher´s exact test.

p-value < 0.05 was considered statistically significant.

### Prevalence of periodontitis in RA cases and controls, in relation to age and gender

**Fig 2:**
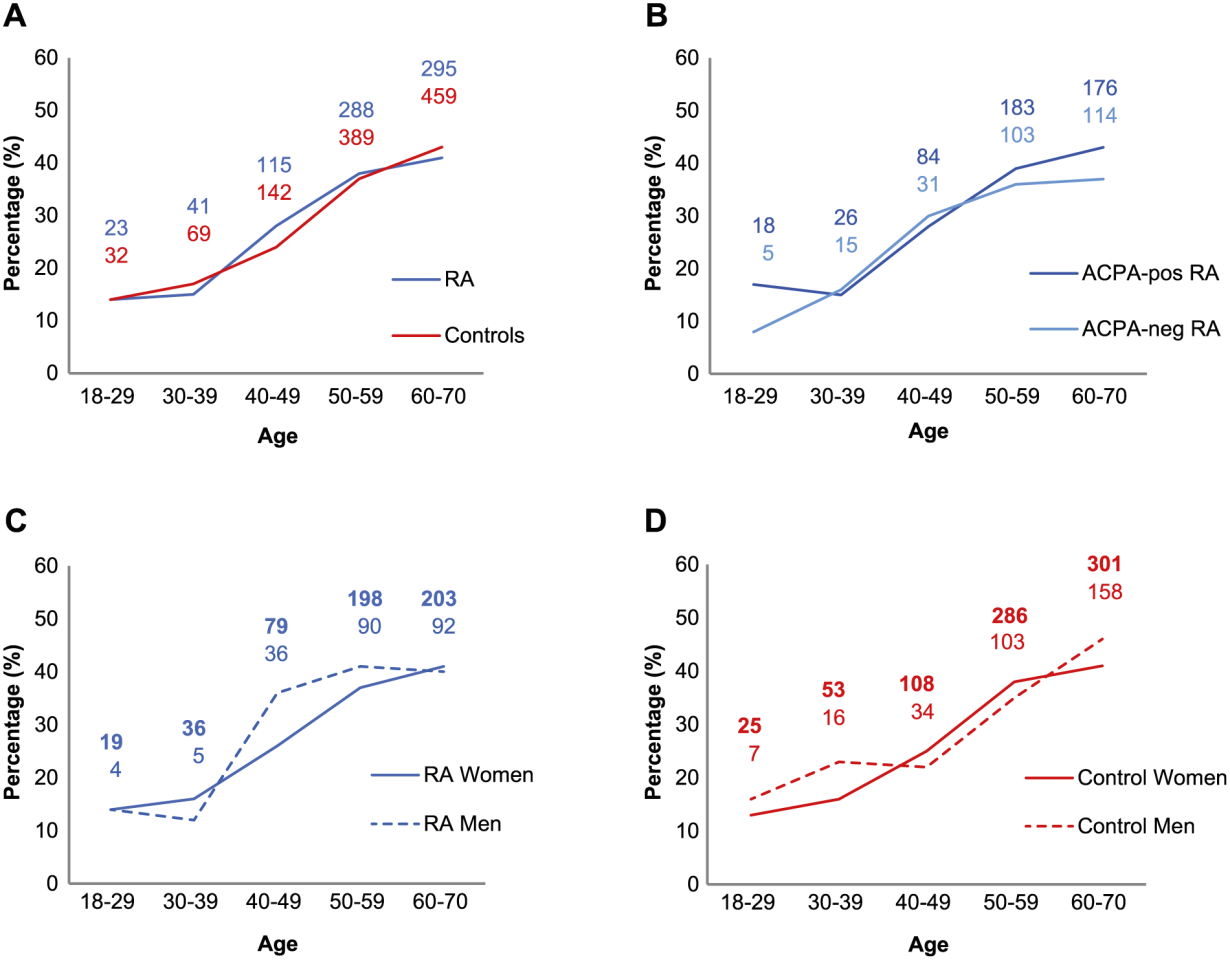
Prevalence of periodontitis in relation to age. Prevalence (%) and numbers of exposed participants with periodontitis in different age groups identified in DHR demonstrating, (**A)** RA cases versus controls, (**B)** ACPA-positive versus ACPA-negative RA patients, (**C)** RA-women versus RA-men, (**D)** control-women versus control-men. Statistical differences were observed with increased age for both RA cases and controls. The differences between the groups were analysed by chi-square test or Fisher´s exact test. p-value < 0.05 was considered statistically significant.

In agreement with previous findings, the prevalence of periodontitis was higher among men compared to women, in both controls (35% versus 31%) [49] and RA cases (36% versus 31%). In order to validate the DHR, we used the known risk factors, smoking and age, for periodontitis [7, 50] to further analyse the association between RA and periodontitis within the EIRA cohort. When dividing EIRA subjects into different age groups, prevalence of periodontitis—as determined based on the diagnostic code for periodontitis—was shown to increase significantly (p < 0.01) by age, in line with previous reports [51], in both RA and controls (Fig 2A) irrespective of ACPA-status or gender (Fig 2B, 2C and 2D). There were, however, no statistical differences in the age groups between RA cases and controls irrespectively of gender (Fig 2A, 2C and 2D), or between ACPA-positive and ACPA-negative RA (Fig 2B).

Since patients with gingivitis do not necessarily also suffer from periodontitis as their gingivitis could be caused simply by poor oral hygiene, and since we are specifically interested in the potential link between RA and periodontitis in the present study, we focused our analyses on the diagnostic codes indicating periodontitis rather than gingivitis.

### Association between smoking habits and periodontitis in RA cases and controls

The risk for periodontitis, as obtained by having at least one of the codes periodontitis, increased risk for periodontitis, peri-implantitis or increased risk for peri-implantitis, increased significantly in current smokers as compared to never smokers (Table 4). This increase was seen both among all RA cases (OR = 1.6, 95% CI: 1.2 to 2.0, p < 0.05) as well as controls (OR = 1.8, 95% CI: 1.5 to 2.2). Also ever-smokers, were shown to have an increased risk for periodontitis irrespective of RA diagnosis (OR = 1.4, 95% CI: 1.2 to 1.7 for RA and OR = 1.3, 95% CI: 1.1 to 1.5 for controls) after adjustments for age, gender and residential area (Table 4). For ex-smokers, the highest OR for periodontitis was observed among RA cases for all (OR = 1.4, 95% CI: 1.1 to 1.7, p < 0.05) and women (OR = 1.5, 95% CI: 1.1 to 1.9, p < 0.05) (Table 4).

**Table 4:** Association between periodontal codes and smoking habits among RA cases and controls, as compared to never smokers, stratified by gender.

Smoking habits	RA cases		Controls	
	Exposed (n)^‡^	OR (95% CI)^†^	Exposed (n)^‡^	OR (95% CI)^†^
**Ex-smokers**				
All	430	1.4 (1.1–1.7)^a^	527	1.1 (0.9–1.3)^a^
Women	291	1.5 (1.1–1.9)^a^	347	1.2 (1.0–1.4)^a^
Men	139	1.2 (0.7–1.8)^a^	180	0.9 (0.7–1.3)^a^
**Ever-smokers**				
All	888	1.4 (1.2–1.7)	1080	1.3 (1.1–1.5)^a^
Women	623	1.4 (1.2–1.8)	768	1.4 (1.2–1.7)
Men	265	1.3 (0.9–1.9)	312	1.0 (0.8–1.4)
**Current smokers**				
All	346	1.6 (1.2–2.0)^a^	382	1.8 (1.5–2.2)
Women	245	1.6 (1.2–2.1)^a^	288	2.0 (1.6–2.6)
Men	101	1.6 (1.0–2.6)	94	1.4 (0.9–2.2)

Results demonstrate RA cases and controls with at least one of the following periodontal diagnostic codes: periodontitis (3043); increased risk for periodontitis (2041); peri-implantitis (3044) and increased risk for peri-implantitis (2051). RA, rheumatoid arthritis.

^‡^Number of exposed RA cases and controls.

^†^Odds ratios (OR) with a 95% confidence interval (95% CI) were calculated by unconditional logistic regression and adjusted for age, gender and residential area.

^a^p-value < 0.05 for association between periodontal codes and smoking habits as compared to never smokers among RA cases and controls, respectively.

## Discussion

In this well-defined population-based EIRA RA case-control study the prevalence of periodontitis, assessed through linkage to the national registry DHR, did not differ between patients with RA and healthy controls, nor between seropositive and seronegative RA. The validation of DHR, reported here for the first time, demonstrated a good validity for diagnosing periodontitis in EIRA subjects with a sensitivity of 77%, a specificity of 71% and a positive predictive value of 89%. In addition, we also confirmed both age and cigarette smoking as risk factors for periodontitis in both RA cases and controls [7, 52], further validating the dataset. Thus, the detailed information on smoking habits, the design of the EIRA cohort with age-, gender- and residential area-matched controls for each RA patient, together with the access to dental records, including x-rays, allowing us to validate the periodontal diagnosis obtained from the DHR database, supports and validates the overall dataset, analyses and conclusion of our study.

In agreement with our findings, several reports, including large population-based [21, 29] and clinical studies [20, 22–26, 53], show no association between RA and prevalence and/or severity of periodontitis. A study investigating 75 Indonesian patients with established RA and 75 matched controls receiving full mouth examination, showed no significant differences in prevalence/severity of periodontitis between the groups [22]. In addition, a Swedish study by Sjöström et al showed even less frequent periodontal breakdown in RA compared to age and sex-matched healthy controls [24]. Moreover, in the Nurses Health Study, the largest prospective study investigating periodontal surgery and/or tooth loss in female nurses (292 with RA and 80 840 controls), followed for more than 12 years, showed no association with RA [21]. Additionally, in a longitudinal study, where 9702 patients (138 with established RA and 433 with incident RA) were followed for 20 years, it was reported that subjects with periodontal disease experienced numerically higher prevalent/incident RA than subjects without periodontitis, although most ORs were non-statistically significant [29]. Conversely, several reports, mostly small clinical studies investigating established RA and systemically healthy controls, have suggested a positive association between RA and periodontitis [12–15, 17, 18, 28, 54, 55]. For example, in a study by de Smit et al, a significantly higher prevalence of moderate and severe periodontitis, diagnosed by using the Dutch periodontal screening index (DPSI), was reported in 95 established RA patients compared to 420 age- and gender matched controls [54]. Moreover, the largest study presented to date, the nationwide linkage study from Taiwan using administrative data on 13 779 newly diagnosed subjects with RA and 137 790 controls, found a weak association (OR = 1.16; 95% CI: 1.12–1.20) between periodontitis and incident RA, although importantly, this analysis was not adjusted for smoking status [27]. The conflicting reports on the association between periodontitis and RA may depend on various factors, including ethnic differences between study populations, different adjustments for confounding variables between studies and substantial differences in disease classification criteria for periodontitis. This suggestion is further supported by Linden et al concluding that existing studies do not provide support for a link between periodontitis and RA since very few studies meet a stringent threshold for periodontitis diagnosis [39]. In addition, although several articles suggest a possible association between periodontitis and RA, recent review reports also conclude that studies with sufficient sample size are needed to ascertain the temporal relationship as well as the biological processes between periodontitis and RA [40, 56, 57].

It has been hypothesized that oral bacterial infection, caused by the periodontitis associated pathogen *P*. *gingivalis*, may be involved in the generation of citrullinated autoantigens and the antibody response by ACPAs [6] which are highly specific and sensitive markers for RA. Based on differences in genetic and environmental risk factors between ACPA-positive and ACPA-negative RA, it has been suggested that different pathogenic pathways underlie seronegative and seropositive RA disease [31]. We therefore also investigated the prevalence of periodontitis with specific focus on ACPA status. In this study, to our knowledge the largest epidemiological investigation of periodontitis in RA in relation to ACPA status performed to date, ACPA status had no effect on the prevalence or the severity of periodontitis. These results are in line with previous reports indicating no association between seropositivity and periodontitis severity [14, 22, 54]. For example, Susanto et al reported no significant differences in ACPA or RF titers between RA patients with no/mild periodontitis as compared to moderate/severe disease in a cohort of 75 Indonesian subjects with RA [22]. Likewise in a Dutch study consisting of 95 subjects with RA, no differences in IgM-RF nor ACPA reactivity were observed when comparing subjects with severe periodontitis with those with moderate disease, as defined by the Dutch periodontal screening index [54]. In contrast, some reports suggest that patients with seropositive RA (ACPA and/or RF) are more likely to have periodontitis or severe periodontitis compared to osteoarthritis subjects or healthy controls [28, 47, 58–60]. For instance, in a cohort of American ACPA-positive RA patients (n = 287) higher mean percentage of sites with alveolar bone loss as compared to patients with osteoarthritis was reported [61]. Similarly, de Pablo et al reported an association between RF-positive RA and edentulism in 103 American patients with RA, after adjustment for age, gender, race/ethnicity and smoking [28]. The conflicting reports on the association between periodontitis and seropositive RA may depend on differences between study cohorts such as type of controls used (osteoarthritis or healthy), ethnicity, relatively small sample size, as well as differences in disease classification criteria for periodontitis.

The current study has addressed the question of association between RA and periodontitis using the well characterized large population based EIRA registry, and the validated DHR database (defining periodontitis according to international consensus criteria [44]). Importantly, most EIRA RA cases identified in DHR would have had RA for a number of years when the linking of registers was performed since EIRA subjects were recruited before 2008, when DHR was initiated. Therefore, we have not investigated whether periodontitis is a risk factor for RA before onset of disease, as periodontitis may have developed after RA onset. Instead, we have investigated whether there is an association between established RA and the existence of periodontitis. The limitations of our study included lack of information about comorbidities in the DHR-cohort and we were not able to investigate for example the effect of diabetes mellitus, reported to be associated with an increased risk of both RA [61] and periodontitis [62]. Furthermore, we had no information regarding the confounding factors such as RA activity or medication including anti-inflammatory agents used for treatment of RA, which have been suggested to decrease the progression of periodontitis [63–65]. Moreover, our results on the prevalence of periodontitis in relation to age included low number of participants in the younger age groups.

In this Swedish EIRA cohort having RA does not associate with increased presence of periodontitis as compared to healthy controls. Since we could not identify an association between RA and periodontitis, or between ACPA/RF-positive RA and periodontitis, the development of periodontitis seems to be independent of RA and/or ACPA and RF status. Although unlikely, we cannot rule out the possibility that certain subsets of RA, not investigated here, may trigger certain subsets of periodontitis, not investigated here. Moreover, whether periodontitis or the differing immune responses to periodontal pathogens are triggering factors for RA cannot be excluded, in particular as our study is based on the identification of periodontitis in a prevalent RA population. Further large prospective epidemiological studies, accounting for RA medication as well as RA disease activity in relation to periodontitis associated pathogens may give new insight into a possible relationship, not identified here.

## Supporting Information

S1 Table Characteristics of the total EIRA study population and the subjects identified in DHR. Results are presented as numbers (%). EIRA, Epidemiological Investigation of Rheumatoid Arthritis; DHR, Dental Health Registry; RA, rheumatoid arthritis; ACPA, anti-citrullinated protein antibody. N/A, not applicable.
(PDF)

S2 Table Validation of the diagnosis of periodontitis obtained from DHR compared to dental records. DHR, Dental Health Registry; CI, confidence interval.
(PDF)

S3 Table EIRA participants, RA cases and controls, without periodontal diagnostic codes (no diagnosis) and participants not identified (missing) in DHR. Results are presented as numbers (%). EIRA, Epidemiological Investigation of Rheumatoid Arthritis; DHR, Dental Health Registry; RA, rheumatoid arthritis.
(PDF)
