# Supplementary material for: Prevalence of Periodontitis in Patients with Established Rheumatoid Arthritis: A Swedish Population Based Case-Control Study
Source: PLoS One. 2016 May 20;11(5):e0155956. doi: 10.1371/journal.pone.0155956 (PMC4874595; doi:10.1371/journal.pone.0155956)
Supplement: S1 Table — Results are presented as numbers (%). EIRA, Epidemiological Investigation of Rheumatoid Arthritis; DHR, Dental Health Registry; RA, rheumatoid arthritis; ACPA, anti-citrullinated protein antibody. N/A, not applicable. (PDF) [file pone.0155956.s001.pdf]

S1 Table. Characteristics of the total EIRA study population and the subjects identified in DHR.

Characteristics		EIRA		DHR	
		RA cases (n = 2740)	Controls (n = 3942)	RA cases (n = 2343)	Controls (n = 3386)
**Gender**					
	Male	764 (28)	1109 (28)	624 (27)	917 (27)
	Female	1976 (72)	2833 (72)	1719 (73)	2469 (73)
**Age**					
	18-29 years	221 (8)	327 (8)	168 (7)	247 (7)
	30-39 years	336 (12)	501 (13)	280 (12)	413 (12)
	40-49 years	486 (18)	687 (17)	408 (17)	597 (18)
	50-59 years	853 (31)	1195 (30)	760 (32)	1056 (31)
	60-70 years	844 (31)	1231 (31)	727 (31)	1072 (32)
**ACPA status**					
	ACPA-positive	1742 (64)	N/A	1469 (63)	N/A
	ACPA-negative	968 (35)	N/A	852 (36)	N/A
**Smoking habits**					
	Never smokers	881 (32)	1664 (42)	777 (33)	1459 (44)
	Ex-smokers	857 (31)	1092 (28)	757 (32)	965 (29)
	Current smokers	733 (27)	763 (19)	576 (25)	588 (18)
	Non-regular smokers	260 (9)	387 (10)	226 (10)	341 (10)
**Education**					
	University degree	625 (23)	1177 (30)	567 (24)	1066 (31)
	No university degree	2112 (77)	2749 (70)	1774 (76)	2307 (68)
**Self-reported periodontitis**					
	Yes	499 (18)	624 (16)	410 (17)	526 (16)
	No	2237 (82)	3314 (84)	1931 (82)	2856 (84)

Results are presented as numbers (%). EIRA, Epidemiological Investigation of Rheumatoid Arthritis; DHR, Dental Health Registry; RA, rheumatoid arthritis; ACPA, anti-citrullinated protein antibody. N/A, not applicable.
