# Supplementary material for: Prevalence of Periodontitis in Patients with Established Rheumatoid Arthritis: A Swedish Population Based Case-Control Study
Source: PLoS One. 2016 May 20;11(5):e0155956. doi: 10.1371/journal.pone.0155956 (PMC4874595; doi:10.1371/journal.pone.0155956)
Supplement: S2 Table — DHR, Dental Health Registry; CI, confidence interval. (PDF) [file pone.0155956.s002.pdf]

**S2 Table. Validation of the diagnosis of periodontitis obtained from DHR compared to dental records.**

| | **%** | **95% CI** |
|---|---|---|
| Sensitivity | 77 | 65-86 |
| Specificity | 71 | 49-87 |
| Positive predictive value | 89 | 78-95 |

DHR, Dental Health Registry; CI, confidence interval.
