# Supplementary material for: Prevalence of Periodontitis in Patients with Established Rheumatoid Arthritis: A Swedish Population Based Case-Control Study
Source: PLoS One. 2016 May 20;11(5):e0155956. doi: 10.1371/journal.pone.0155956 (PMC4874595; doi:10.1371/journal.pone.0155956)
Supplement: S3 Table — Results are presented as numbers (%). EIRA, Epidemiological Investigation of Rheumatoid Arthritis; DHR, Dental Health Registry; RA, rheumatoid arthritis. (PDF) [file pone.0155956.s003.pdf]

S3 Table. EIRA participants, RA cases and controls, without periodontal diagnostic codes (no diagnosis) and participants not identified (missing) in DHR.

**Diagnosis**	**RA cases**		**Controls**	
	**No diagnosis**	**Missing**	**No diagnosis**	**Missing**
Gingivitis	1537 (56)	414 (15)	2170 (55)	572 (15)
Periodontitis	1567 (57)	406 (15)	2268 (58)	576 (15)
Increased risk periodontitis	1720 (63)	418 (15)	2421 (62)	582 (15)
Peri-implantitis	2202 (81)	424 (16)	3203 (81)	592 (15)
Increased risk peri-implantitis	2294 (84)	424 (16)	3322 (84)	593 (15)

Results are presented as numbers (%). EIRA, Epidemiological Investigation of Rheumatoid Arthritis; DHR, Dental Health Registry; RA, rheumatoid arthritis.
